# Langerhans cell histiocytosis presenting as eosinophilic granuloma of the bilateral forearms in an 8-year-old girl: a case report

**DOI:** 10.1186/s13256-019-2011-1

**Published:** 2019-03-19

**Authors:** Salahoudine Idrissa, Hind Cherrabi, Boubacar Efared, Kassim Sidibé, Karima Attaraf, Lamiae Chater, Abderahmane Afifi

**Affiliations:** 1 Department of Paediatric Surgery, University Hospital of Hassan II, Fez, Morocco; 2 Department of Pathology, University Hospital of Hassan II, Fez, Morocco; 3 Department of Radiology, University Hospital of Hassan II, Fez, Morocco; 4 University Sidi Mohamed Ben Abdellah, BP 1893; Km 2.200, Sidi Harazem Road, 246 Avenue Ibn el Khatib, Immeuble 46, Lotissement Ghazali, Quartier elAzhar, 30000 Fez, Morocco

**Keywords:** Langerhans cell histiocytosis, Pediatric bone lesion, Forearm

## Abstract

**Background:**

Langerhans cell histiocytosis previously known as histiocytosis X is a rare disease of children and young adults with a very broad clinical spectrum. In children, its annual incidence is estimated between 0.2–0.5 per 100,000.

**Case representation:**

An 8-year-old Moroccan girl with no known personal or family history presented to our institution with painful swelling of both forearms. An X-ray and magnetic resonance imaging were inconclusive. We then performed a biopsy curettage (of her left forearm). Microscopic analysis followed by immunohistochemical analysis disclosed a diagnosis of Langerhans cell histiocytosis. No chemotherapy was necessary. Clinical and radiological improvement was achieved after 6 months.

**Conclusion:**

The particularity of this observation is the bilaterality of the lesion on both forearms and it has not previously been reported. Langerhans cell histiocytosis should be included in the differential diagnosis of osteomyelitis and Ewing’s sarcoma.

## Introduction

Langerhans cell histiocytosis (LCH) is a rare disorder characterized by a proliferation of cells causing local or systemic effects [1]. LCH includes the clinical entities of eosinophilic granuloma (EG), Letterer–Siwe disease, and Hand–Schuller–Christian disease [2, 3]. EG is the most common form of LCH, and involves single or multiple bones [3]. The disease is characterized by proliferation of the Langerhans cells that usually causes pain and adjacent soft tissue swelling [4]. It is a relatively uncommon disease suffered by infants and children [5]. The involvement of both forearms is extremely rare and not previously reported to the best of our knowledge; it is this rare form of LCH that we report in this observation.

## Case representation

**Fig. 1:**
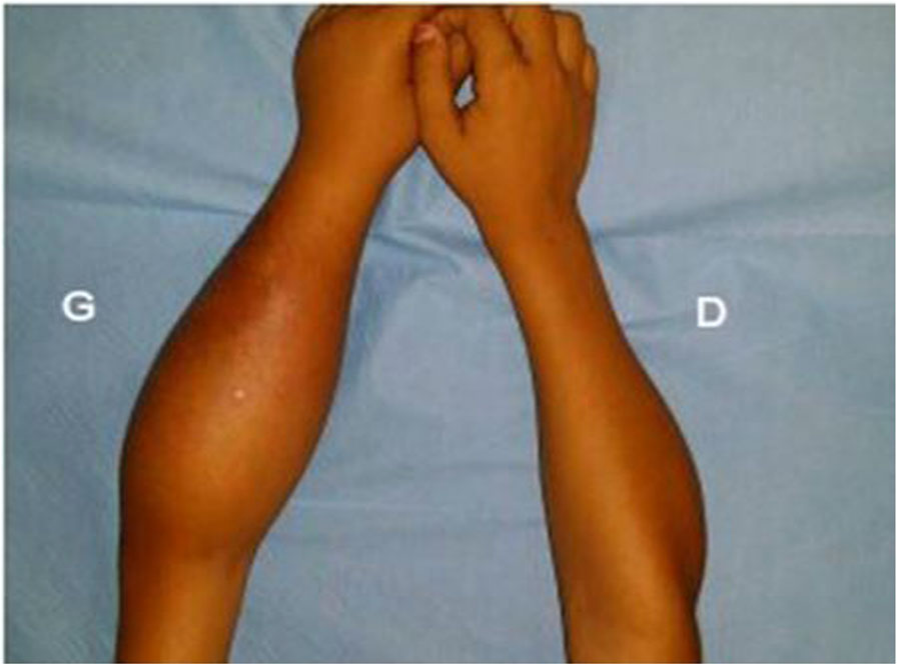
Physical examination. Swelling in the upper one-third of the right forearm (*D*) and the upper two-thirds of the left forearm with inflammatory signs at this level (*G*)

**Fig. 2:**
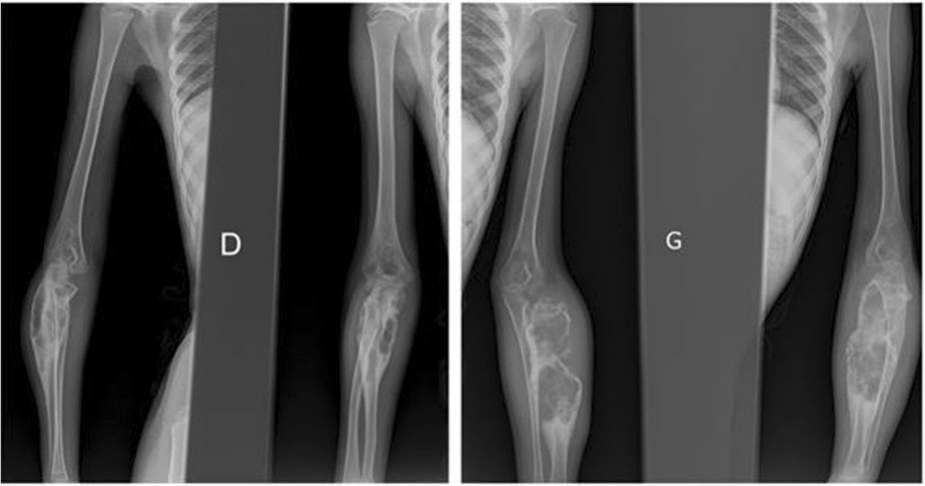
Plain radiographs. *D*) Right forearm: osteolytic lesion of the upper one-third of the right ulna. *G*) Left forearm: osteolytic lesion of the upper two-thirds of the left radius

**Fig. 3:**
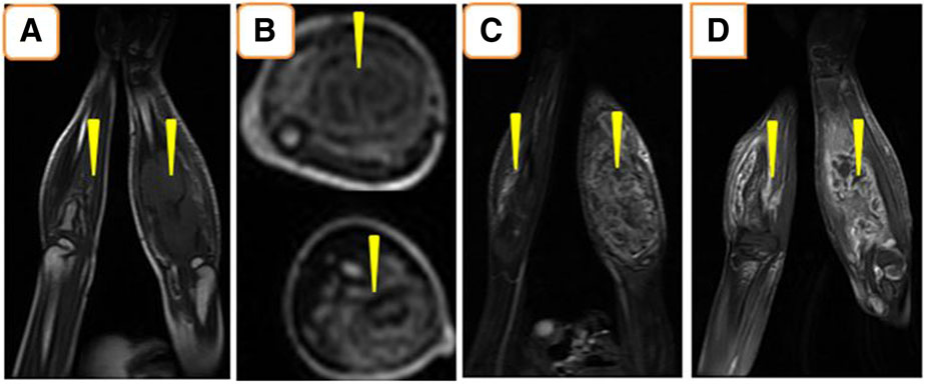
Magnetic resonance imaging findings. An osteolytic lesion mass of the upper one-third of the right ulna and the upper two-thirds of the left radius. The mass invading the articulation of the elbows whose matrix is in hyposignal T1 (**a**), hypersignal T2 (**b**), and short T1 inversion recovery (**c**), containing septa and enhanced annularly after injection of gadolinium (**d**)

**Fig. 4:**
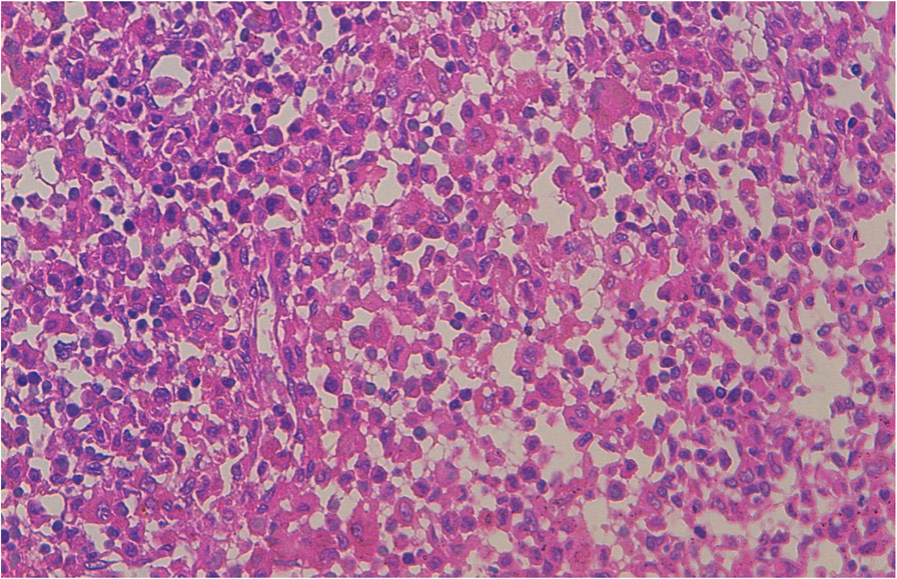
Histopathologic examination. Proliferation of histiocytes with an infiltration of eosinophils

**Fig. 5:**
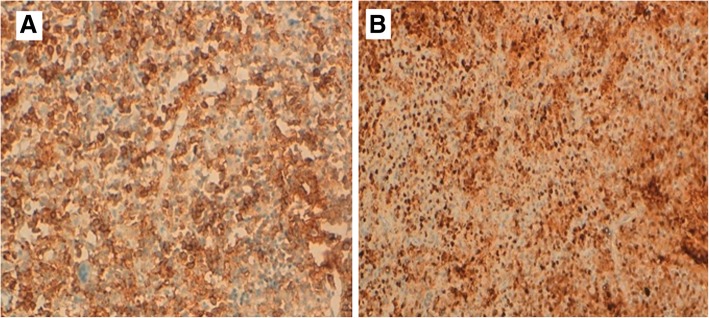
Immunohistochemistry. S-100 protein (**a**), and for CD-1a (**b**)

**Fig. 6:**
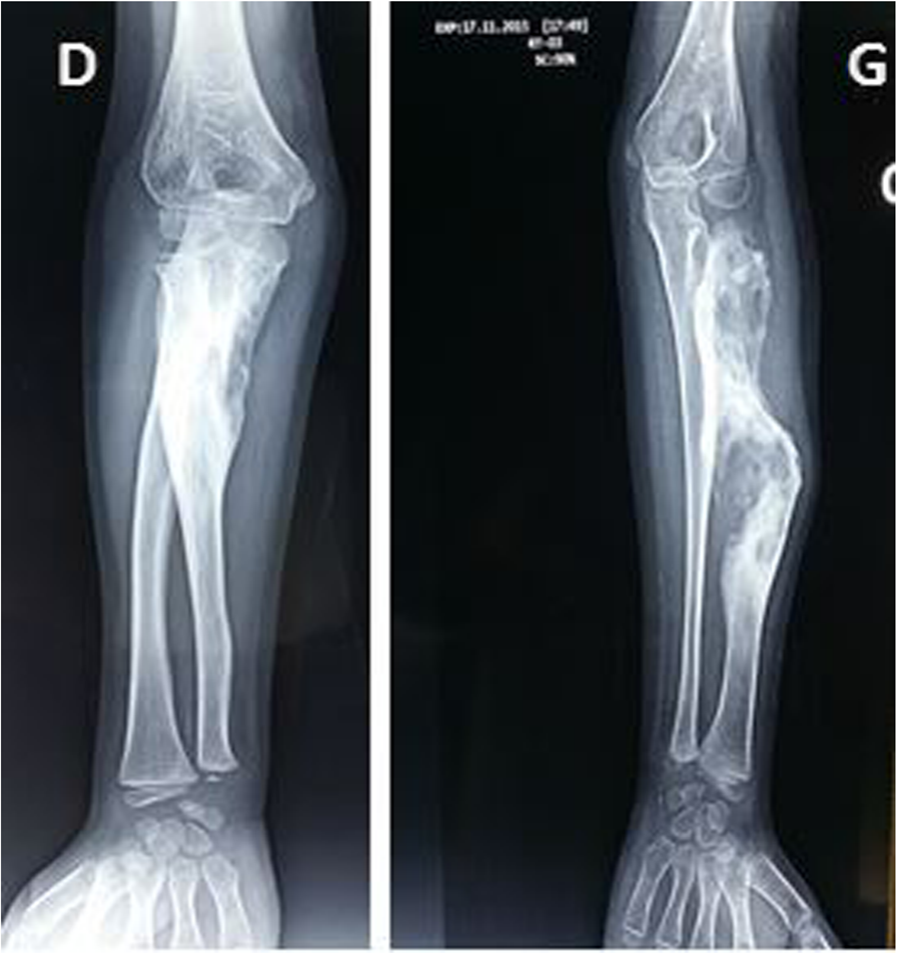
X-ray of the patient 6 months later (*D*, *right*; *G*, *left*). The improvement is clear

An 8-year-old Moroccan girl presented to our institution with painful swelling of both forearms which initially appeared on her left forearm and 6 months later on her right forearm. Her family history and medical history were unremarkable. Given the exacerbation of the pain she consulted a doctor who obtained plain radiographs and noted a lesion in both forearms. She was then referred to us for further evaluation. She reported that despite daily use of nonsteroidal anti-inflammatory medications and narcotic analgesics, the pain in her forearms continued to progress. On physical examination she had no fever and had a good general condition. She presented a swelling in the upper third of her right forearm and the upper two-thirds of her left forearm with inflammatory signs (Fig. 1). Laboratory studies found a moderate anemia (hemoglobin at 10 g/dL) and a white blood cell count of 11,210/μL with 80% neutrophils. Her C-reactive protein level was 60 mg/L. We obtained plain radiographs (Fig. 2) that showed: an osteolytic lesion of the upper one-third of the right ulna and osteolytic lesion of the upper two-thirds of the left radius. On both forearms, we did not note mineralized matrix production, but a cortical breakthrough and internal trabeculations were present. We therefore performed magnetic resonance imaging (MRI) (Fig. 3) which showed: an osteolytic lesion mass (arrow) of the upper one-third of her right ulna and the upper two-thirds of the left radius. The mass was invading her elbow joint whose matrix was in hyposignal T1 (Fig. 3a), hypersignal T2 (Fig. 3b), and short T1 inversion recovery (STIR) (Fig. 3c), containing septa and enhanced annularly after injection of gadolinium (Fig. 3d). The lesion began in the diaphysis and crossed the physis. A soft tissue mass and cortical breakthrough were noted. A soft tissue edema was also seen. A biopsy curettage of her left forearm was done. A histopathologic examination revealed a proliferation of histiocytes with an infiltration of eosinophils (Fig. 4). These histiocytes were positive for S-100 protein (Fig. 5a) and for CD-1a (Fig. 5b). No chemotherapy was necessary. Our patient’s symptoms disappeared after a short (5 days) period of nonsteroidal anti-inflammatory therapy. A repeat X-ray was obtained (Fig. 6) and showed a partial improvement of the osteolysis. She remained asymptomatic after 6 months.

## Discussion

LCH is a rare disease with an incidence of 0.2–0.5 per 100,000 children per year [6]. The disease involves typically patients aged from 5 to 15 years in approximately 90% of cases, with a slight male predominance [6]. To the best of our knowledge, a bilateral localization of LCH on both forearms has not been reported. The most frequent site of EG is the skull followed by the femur, then the ribs. Localization in the forearm is approximately 1.5% [7]. The most common complaint is the pain, often worse at night [8]. However, a fracture, a swelling, a deformity, and a soft tissue component are also encountered. The lesions caused by LCH are often located on the diaphysis, the metaphysis, or extend to the physis and epiphysis of the long bone [9]. Cortical thinning, intracortical tunneling, and medullar widening are often found on radiography. The periosteal reaction, while less common, may be present at an early phase of the disease, giving a more aggressive and pseudomalignant appearance [9]. The most common lesions on MRI are perilesional bone marrow edema, periosteal reaction, endosteal scalloping, and postcontrast enhancement with or without soft tissue mass [10]. The differential diagnoses include aneurysmal bone cyst, osteomyelitis, Ewing’s sarcoma, osteoblastoma, Gaucher’s disease, acute leukemia, and metastatic tumor [11]. The diagnosis of EG is based on histological and histochemical identification. The therapeutic modalities include observation for spontaneous resolution, biopsy, curettage with or without bone grafting, local steroid injection, anti-inflammatory drugs, bisphosphonates, radiotherapy, chemotherapy, and immunotherapy [6]. The results of treatment of solitary lesions are always satisfactory. In contrast, multifocal and multisystem types of LCH are generally treated with chemotherapy in combination with other therapeutic modalities [5].

## Conclusion

LCH is a rare disease in children with a very broad clinical spectrum. The particularity of this observation is the bilaterality of the lesion on both forearms and it has not previously been reported to the best of our knowledge. LCH should be included in the differential diagnosis of osteomyelitis and Ewing’s sarcoma.
